# Eye Movement Desensitization and Reprocessing Versus Treatment as Usual in the Treatment of Depression: A Randomized-Controlled Trial

**DOI:** 10.3389/fpsyg.2018.01384

**Published:** 2018-08-14

**Authors:** Michael Hase, Jens Plagge, Adrian Hase, Roger Braas, Luca Ostacoli, Arne Hofmann, Christian Huchzermeier

**Affiliations:** ^1^Lüneburg Center for Stress Medicine, Lüneburg, Germany; ^2^Department of Psychosomatics and Psychotherapy, Diana Klinik, Bad Bevensen, Germany; ^3^School of Sport, Rehabilitation and Exercise Sciences, University of Essex, Colchester, United Kingdom; ^4^Department of Psychiatry and Psychotherapy, Central Hospital of the German Armed Forces, Koblenz, Germany; ^5^Clinical Psychology and Psychosomatics Service, University Hospital San Luigi Gonzaga, University of Turin, Turin, Italy; ^6^EMDR Institute Germany, Bergisch Gladbach, Germany; ^7^Center for Integrative Psychiatry, Institute of Sexual Medicine, Forensic Psychiatry and Psychotherapy, University of Schleswig-Holstein, Kiel, Germany

**Keywords:** depression, eye movement desensitization and reprocessing, randomized-controlled trial, Beck Depression Inventory, symptom checklist 90-revised

## Abstract

Eye movement desensitization and reprocessing (EMDR) is a well-established treatment for post-traumatic stress disorder. Recent research suggested that it may be effective in treating depressive disorders as well. The present study is part of a multicenter randomized-controlled trial, the EDEN study, in which a homogenous group of 30 patients was treated to test whether EMDR plus treatment as usual (TAU) would achieve superior results compared to TAU only in a psychosomatic-psychotherapeutic inpatient treatment setting. Both groups were assessed by the Beck Depression Inventory-II (BDI-II) and the Global Severity Index and depression subscale of the Symptom Checklist 90-Revised. The EMDR + TAU group improved significantly better than the TAU group on the BDI-II and Global Severity Index, while a marginally significant difference favoring the EMDR + TAU group over the TAU group was found on the depression subscale. In the EMDR + TAU group, seven out of 14 patients improved below nine points on the BDI-II, which is considered to be a full remission, while four out of 16 in the TAU group did so. These findings confirm earlier suggestions that EMDR therapy may provide additional benefit in the treatment of depression. The present study strengthens the previous literature on EMDR therapy in the treatment of depression due to the randomized-controlled design of the EDEN study.

## Introduction

According to the often-considered study of the World Health Organization (World Health Organization [WHO], 2012), depressive disorders belong to the most prevalent and disabling diseases of all: At least 350 million people are affected by depressive disorders worldwide, almost one million of which commit suicide every year (Murray and Lopez, 1996; Greden, 2001). Due to their frequency and severity, depressive disorders thereby belong to the biggest worldwide challenges of the psychiatric profession.

Treatment options for depressive spectrum disorders are partially favorable, but also partially problematic. Although pharmacological as well as psychotherapeutic treatment approaches are available, incomplete remission and high long-term relapse rates remain for many patients. Research has shown that psychotherapeutic interventions can be helpful – not only in mild and moderate depression, but also in cases of severe and chronic depression (Nemeroff et al., 2003). In a meta-analysis by Vittengl et al. (2007), however, 29% of those who responded to acute-phase cognitive-behavioral therapies relapsed after 1 year, and 54% relapsed after 2 years. Furthermore, the available pharmacological treatments for depressive disorders are associated with several issues. Although these treatments improved in the last 20 years, the optimism associated especially with recent antidepressants like the SSRI class (e.g., Fluoxetine) has faded due to meta-analyses on antidepressant pharmacotherapy showing only a slight advantage over placebo. The greatest treatment success was shown in a study with predominantly severe depression (Fournier et al., 2010), wherein antidepressant treatment was often associated with side effects (e.g., weight gain and other problems lasting over time; Hirschfeld, 2003; Kripalani et al., 2007; Reid and Barbui, 2010). Though a systematic review based on 31 randomized studies has shown that relapse rates may be reduced by 50% with antidepressant medication (of all classes; Geddes et al., 2003), the very high likelihood of depressive relapses often leads to lifelong medication. Incidentally, depressive symptoms remaining after treatment and the degree of treatment resistance relating to the previous depressive episode are considered risk factors for a relapse (Reid and Barbui, 2010). Additionally, it is noteworthy that between 10 and 20% of depressive episodes become chronic or are considered treatment resistant to standard depression treatments. Furthermore, the danger of relapsing increases not only when specific personality traits, dysfunctional beliefs, and/or cognitive schemas are present, but also in response to experience of trauma or critical life events. In summary, the current treatment effects and especially the high relapse rates in acute depressive episodes are unsatisfactory. However, adjunctive psychotherapeutic treatment has been found to reduce the risk of relapse by 22% when compared with pharmacological antidepressant treatment alone (Vittengl et al., 2007).

In order to further improve treatment effects and lower relapse rates, it may be necessary to put greater emphasis on the importance of traumatic experiences and adverse life events for the development and progression of depression. For instance, it is a well-known clinical observation that depression may be triggered and maintained by stressful life events. Recent research indicates that chronic and acute stressors like traumatic experiences and other adverse life experiences like loss, hurt, and humiliation can trigger depressive disorders (Heim and Nemeroff, 2001; McFarlane, 2010). Especially so-called primary episodes are often closely linked with a specific psychosocial stressor, while later depressive episodes may be triggered by far smaller events or even come about without any noticeable stressor (Post, 1992). Risch et al. (2009) could also show the strong influence of stressful life events in a large meta-analysis: According to their analysis, stressful life events are the only risk factor to be significantly correlated with the onset of depression. For instance, a serotonin transporter gene polymorphism as a neurobiological vulnerability factor alone, or in combination with adverse life events, did not significantly correlate with the occurrence of depressive episodes. Similarly, a large case-control study found an association in which the risk for depression doubled when violent victimization was experienced in early life (Wise et al., 2001). Furthermore, Mandelli et al. (2015) found that childhood emotional abuse and neglect correlate with the highest risk for experiencing depressive disorders in adulthood, even when compared to other forms of childhood trauma like physical abuse or sexual abuse. Some researchers have also brought up the notion that adverse life events could have similarly severe effects on depression as the far more stressful traumatic experiences described in the type A criterion definition of the DSM (Gold et al., 2005). This is also supported by data from a survey of 832 people (Mol et al., 2005), which showed that stressful life events can generate at least as many post-traumatic stress disorder (PTSD) symptoms as classical traumatic events according to the type A criterion. For stressful life events dating up to 30 years back, the PTSD symptomatology was more pronounced than for traumatic events that corresponded with the type A criterion.

In light of the previously presented research, it makes sense to develop complementary therapy strategies. Eye movement desensitization and reprocessing (EMDR) therapy is a promising candidate for such a complementary strategy that could provide an additional benefit in the treatment of depression. The treatment was first developed by Shapiro (1989, 2001) after a serendipitous observation of the relaxing effect of horizontal saccadic eye movements was initially used to treat PTSD, and has proven its effectiveness in this field (Bisson and Andrew, 2007). It targets memories of critical life events as well as traumatic experiences and enables the psychotherapeutic focus on maladaptive cognitive patterns. Though Shapiro (1989, 2001) at first observed the therapeutic effectiveness of EMDR in PTSD, she increasingly observed effects on other symptoms (e.g., anxiety), which led to EMDR being used to treat other disorders that may also be based on, or exacerbated by, unprocessed and maladaptively stored memories of stressful life events. The main principle of EMDR therapy thus is the reprocessing of maladaptively stored (pathogenic) memories that produce symptoms when activated by sensory cues (Centonze et al., 2005). The effectiveness of EMDR has also been shown by neurobiological research showing a normalization of brain activity in the sense of more adaptive information processing (AIP) after EMDR treatment (Pagani et al., 2013). The reprocessing part of EMDR is initiated and maintained by bilateral stimulation – mainly through eye movements, but alternatively also through bilateral alternating auditory or tactile stimulation.

While its efficacy as a PTSD treatment has been well-researched, the effectiveness of EMDR in the treatment of depression has only recently begun to receive systematic research attention (Hofmann et al., 2014; Hase et al., 2015). Previously, what stood out in studies of PTSD was that EMDR concomitantly improved comorbid depressive symptoms along with the main PTSD symptomatology. For instance, several case reports showed that depressive patients could be successfully treated with either EMDR therapy alone or with EMDR therapy as an adjunct to other approaches (Manfield, 1998; Tinker and Wilson, 1999; Sun et al., 2004; Broad and Wheeler, 2006; Shapiro and Grand, 2009; Rosas Uribe et al., 2010; Grey, 2011). For instance, two adolescents with major depression were successfully treated with EMDR therapy only (three and seven sessions, respectively) and showed stable improvements in a 3-month follow-up examination (Bae et al., 2008). In both cases, EMDR was successfully applied in the processing of relationship losses or changes. Such events (relationship losses or negative changes) also seem to be a specific risk factor for depressive disorders. In a large retrospective study, losses, separation events, and humiliating events were strongly associated with an increased risk for depressive episodes (Kendler et al., 2003). Going beyond case reports, van der Kolk et al. (2007) conducted a randomized clinical trial comparing the effectiveness of fluoxetine with EMDR treatment and placebo in a PTSD population and found the EMDR group to have significantly lower depression scores than the fluoxetine group. This led them to conclude that once “…the trauma is resolved, other domains of psychological functioning appear to improve spontaneously” (van der Kolk et al., 2007, p. 8). This result had previously been found by similar controlled studies, such as a study of Power et al. (2002) in which PTSD patients were either treated with cognitive behavioral therapy (CBT) or with EMDR (there was a wait list control group). Both treatment groups experienced significant improvements in PTSD and comorbid depression symptoms, which also showed at 6-month follow-up.

Out of these research results, the idea emerged that EMDR therapy may be a helpful adjunct treatment in the treatment of depression. To test this, a larger study investigated whether different results may be obtained in depressive patients without an explicit trauma history when adding additional EMDR therapy in comparison with CBT treatment (Hofmann et al., 2014). In this study with a group of 42 depressive patients, one group was treated with CBT (21 patients) and the other one with CBT + EMDR (seven additional EMDR sessions). The CBT + EMDR group showed more complete remissions and a greater reduction in Beck Depression Inventory (BDI) scores than the CBT only group. In another matched-pairs study in a clinical setting (Hase et al., 2015), 11 out of 16 patients (68%) in the EMDR group showed a complete remission of depressive symptomatology at the end of treatment. The EMDR group also showed a greater reduction of depressive symptoms than the CBT only group. However, it should be noted that the generalizability of the findings was limited due to the small sample and lack of a randomized-controlled design.

On the whole though, these previously mentioned studies provided first empirical indications that EMDR therapy may have significant positive effects in the treatment of depressive episodes and recurrent depressive disorders. This provided an incentive to conduct higher-quality clinical studies like the present study, which presents the first randomized-controlled clinical trial looking at adjunctive EMDR therapy in the treatment of depression. In this study, we proposed the following hypotheses:

(1) EMDR therapy produces an additional benefit over treatment as usual in the treatment of patients with acute depressive episodes.
(2) EMDR therapy increases the proportion of complete remissions in the treatment of patients with acute depressive episodes.

## Materials and Methods

### Study Setting and Study Participants

The study was part of a Europe-wide multicenter study (EDEN) examining the effects of EMDR in the treatment of depressive disorders. The aim of the study was to replicate previous results showing that EMDR contributes to the improvement of depressive disorders in a larger patient group. The study also aims to show, via the analysis of follow-ups recorded in the EDEN study, whether the number of depressive relapses can be reduced. The study was carried out in accordance with the recommendations of the ethical guidelines of the Declaration of Helsinki with written informed consent being obtained from all participants. The protocol was approved by the ethics committee of the University of Kiel.

**Table 1** presents sample demographic information. The sample consisted of 30 inpatients of a psychiatric and psychosomatic rehabilitation clinic receiving treatment for a moderate to severe depressive episode. The treatment as usual (TAU) group comprised 16 patients and the EMDR + TAU group comprised 14 patients. Included ICD-10 diagnoses were F32.1 (three in TAU, four in EMDR + TAU group), F33.0 (one in TAU, none EMDR + TAU group), F33.1 (ten in TAU, nine in EMDR + TAU group), F33.2 (one in TAU, one in EMDR + TAU group), and F33.4 (one in TAU, none in EMDR + TAU group). All participants were patients (privately insured through the German Armed Forces) in the department of psychosomatic medicine and psychotherapy at the Diana rehabilitation center clinic, Bad Bevensen, Germany. In the context of standard admission procedures with clinical anamnesis and gathering of existing psychopathology according to AMDP, the diagnosis of depression (ICD-10 F32.x and F33.x) was made. Patients that were eligible for the study were extensively informed about the chances and risks of an additional treatment with the EMDR method and gave their written informed consent. In the case of consent, they were added to the EDEN database and concomitantly randomized in one of the two treatment groups (see below). The EMDR treatment was administered according to manualized EMDR procedures (Shapiro, 2001) and the EDEN study protocol (Hofmann et al., 2016).

**Table 1:** Sample demographics by treatment group.

		TAU (%)	EMDR + TAU (%)	Sig.^1^
Children	None	11 (69)	7 (50)	0.30
	One or more	5 (31)	7 (50)	
Education	Post-secondary	5 (31)	4 (29)	0.93
	Post-secondary (vocationally restricted)	1 (6)	1 (7)	
	Secondary	9 (56)	7 (50)	
	Lower secondary	1 (6)	2 (14)	
Marital status	Unmarried	9 (56)	3 (21)	0.13
	Married	6 (38)	8 (57)	
	Divorced/Separated	1 (6)	3 (21)	
Sex	Male	14 (88)	13 (93)	1.00
	Female	2 (13)	1 (7)	
Age		39.23 (10.02)	40.32 (9.25)	0.78

*Frequencies with corresponding percentages (rounded to the closest integer) given in parentheses. For Age, mean and standard deviations (in parentheses) are provided instead of frequencies and percentages. ^1^The p-value for the Children by Treatment Group comparison was derived from a chi-squared test. The p-values for Education by Treatment Group and Marital Status by Treatment Group were derived from Fisher’s exact test as expected cell totals below five occurred in the respective contingency tables. The p-value for Age by Treatment Group was derived from an independent-samples t-test.*

Inclusion criteria were: The presence of a depressive episode or a recurrent depressive disorder according to clinical diagnostic findings, at least mild depression with a BDI-score of more than 12, and current psychopharmacological antidepressant treatment.

Exclusion criteria were: Acute suicidality, detected comorbidities like, for example, personality disorders or addiction disorders, psychotic symptomatology, complex PTSD, and a pronounced dissociative symptomatology (detected with scores of >25% in the standardized questionnaire “DES-II,” disorders of the eye (e.g., acute retinal detachment or recent eye surgery), or simultaneously running judicial trials or statutory pension insurance scheme applications to prevent external obstacles to a successful treatment. The only dropout criteria were the emergence of acute suicidality or the withdrawal of informed consent.

In the early diagnostics, complex PTSD was selected as an exclusion criterion to minimize risks and side effects in the study. As was shown in multiple studies (Frustaci et al., 2010; Rosas Uribe et al., 2010; Hofmann et al., 2014), EMDR treatment is well tolerated when controlling for contraindications. All study participants were offered the opportunity to receive up to two outpatient follow-up care visits in the rehabilitation center, if needed.

### Procedure of Data Collection

The beginning of the data collection started with the admission to the inpatient psychosomatic treatment in the department of psychosomatic medicine and psychotherapy of the Diana clinic. For randomization, the EDEN database was used. The EDEN database was developed for the EDEN study, which has been running since 2012 as a multicenter study in six centers in four European countries (Italy, Germany, Spain, and Turkey). In this study, the EDEN database randomized participants into the control group (TAU) and the treatment group (TAU + EMDR). The project also focuses on the research question of whether the number of relapses may be reduced by EMDR treatment through a planned follow-up taking place 1 and 2 years after treatment. The measurements with the instruments described below were partly taken on a weekly basis (BDI), and partly only at beginning and end of treatment (SCL-90-R, see below). An assessor who was blind to participants’ assigned conditions administered all of these measurements, which were computer-based.

### Beck Depression Inventory II

The Beck Depression Inventory II (BDI-II; Hautzinger et al., 2006) is a self-report instrument to assess of the severity of depressive symptomatology and its change in response to treatment (the study comparison considers admission and end-of-treatment scores). The sum score of this test can range from 0 to 63 points. If the patient checks multiple answer options in one item, the highest selected number of points will count toward the sum score. A score of less than nine points falls into the normal range. Scores between nine and 13 indicate a minimal severity of depressive symptoms. Scores between 14 and 19 indicate a mild depressive disorder. Scores of 20 or higher are considered clinically relevant, with scores between 20 and 28 indicating a moderate depressive disorder, and scores higher than 29 indicating severe depression. The BDI-II maps a wide spectrum of depressive symptomatology (Beck et al., 1961) and features high reliability and validity. Moderate to high correlations show concurrent validity with different depression scales. Albeit no exact value is listed for the diagnosis of a depressive disorder, a comparing statement is possible.

### SCL-90-R

The Symptom-Check-List 90 Items Revised-Version (SCL-90-R; Derogatis et al., 1973) is an instrument to record subjective impairment due to physical and mental symptoms within a time frame of 7 days. The test is also suitable for checking the course of a disorder. The Global Severity Index (GSI) gives an indication of the overall burden for any given patient with symptoms on all scales. Of the nine factorial scales, the depression subscale was additionally used in the study. Measurements are given in the form of standardized *t*-values here. They fall within the normal population when they are between 40 and 60. The mean score is thus 50 (*SD* = 10). Values of 60–64 are considered to be slightly elevated, 65–69 considerably elevated, 70–74 strongly elevated, and 75–80 very strongly elevated.

### Treatment Methods

The TAU group comprised 16 patients with depressive symptomatology satisfying the in- and exclusion criteria listed above. These patients were treated in the usual clinical setting with a psychodynamic or behavioral group therapy (participation twice or 90 min per week) and a standard individual therapy. They all received antidepressant medication (which is listed as an inclusion criterion above).

The EMDR + TAU group comprised 14 patients that were treated in the same clinical setting as the TAU group, receiving the same TAU treatment including antidepressant pharmacotherapy. In addition, it was planned to process one unprocessed memory with EMDR per week, which requires one to two sessions per week. It is important to highlight that the EMDR group did not receive as many standard individual therapy sessions as the TAU group due to the administration of EMDR. The EMDR + TAU group received between 4 and 12 EMDR sessions in total (*M* = 8.5, *SD* = 2.41).

The so-called EMDR standard protocol is split into eight treatment phases. In the application of the EMDR therapy, the work is usually conducted in the three domains of past, present, and future. In the domain of the past, dysfunctionally stored pathogenic memories are being reprocessed. In the domain of the present, experience-related nightmares, triggers, and also abnormal behaviors are targets of the EMDR treatment. In the domain of the future, the therapy targets the change of avoidance behavior and the development of respective behavioral alternatives, and anxiety concerned with a possible future depressive relapse. In all areas, dysfunctionally stored and unprocessed information is the target of the EMDR treatment. The eight treatment phases according to the EMDR standard protocol are ideally structured as:

Phase 1: History and Treatment Planning – In phase 1, the precise anamnesis and clinical history are recorded. In doing this, it is especially important to give an indication for or against the EMDR method, which also means it is about the exclusion of contraindications. This is also done with the help of specific test diagnostics.
Phase 2: Preparation – In phase 2, a precise treatment plan is made and the patient receives extensive information about the method. If necessary, the learning of relaxation or imaginative techniques, as well as pharmacological treatment may take place at this point to ensure sufficient stabilization.
Phase 3: Assessment – In phase 3, the dysfunctional stressful memory in question is activated in its affective, sensory, and cognitive components. In doing this, the entire pathogenic memory is activated through the controlled and fractional activation of partial networks (according to LeDoux, 2001).
Phase 4: Desensitization – The method then proceeds to the central phase of the processing work, where the patient connects with the memory. At the same time, bilateral stimulation is applied here, mostly by therapist-guided eye movements. From here on, the process typically proceeds idiosyncratically and individually. The quick associative succession of changing affective and sensory impressions and thoughts is characteristic here. This often leads to a notable relief in the patient, although intensively experienced affects or physical symptoms (affective or somatic reactions) may also be registered in the meantime. The gradual relief experienced in this offers a great advantage for the processing in the patient. The pressure generated by the mobilized memory material remains well-manageable therapeutically.
Phase 5: Installation – Once the degree of stress has sufficiently decreased in phase 4 and the positive cognition that was identified in phase 3 has clearly gained strength (as checked by the therapist), a strengthening of the positive cognition is enforced by bilateral stimulation. In doing so, it appears to be sustainably cognitively installed.
Phase 6: Body Scan – The body scan serves to search for potentially persisting sensory memory. In case any of them are encountered, they will be reprocessed by adding bilateral stimulation.
Phase 7: Closure – Since the experience that the patient makes from phase 4 to phase 6 is typically very impressive, it is extensively discussed with the therapist afterwards. The possibility of reprocessing material that surfaced during the session or was not completely processed is also presented to the patient.
Phase 8: Re-evaluation – This phase serves as a platform for patient feedback about changes after previous sessions.

### Statistical Analysis

An analysis of covariance (ANCOVA) was run for BDI-II, SCL-90-R GSI, and SCL-90-R depression subscale scores as dependent variables with treatment group as the main independent variable. The analyses controlled for type of diagnosis (single/recurrent depression), patient age, total number of days in treatment, and the score on the respective dependent variable at the beginning of treatment. Interactions between treatment group and the covariates type of depression, patient age, and total number of days in treatment were included in the model. A simple contrast with the TAU group as the reference group was used to examine potential differences between the two groups.

## Results

There were no statistically significant differences between the scores on the recorded outcome measures (BDI-II, SCL-90-R depression subscale, and SCL-90-R GSI) and age between the two groups at the beginning of treatment. **Table 2** presents descriptive statistics for said outcome measures and patient age at the beginning and at the end of treatment, grouped by treatment.

**Table 2:** Mean and standard deviations for both groups.

		Baseline	End of treatment
BDI-II	TAU	23.02 (5.86)	16.59 (11.35)
	EMDR + TAU	22.43 (8.75)	12.21 (11.23)
SCL-90R depression subscale	TAU	72.06 (6.53)	65.07 (9.23)


	EMDR + TAU	69.79 (8.20)	59.71 (13.71)
SCL-90R GSI	TAU	70.63 (6.00)	62.40 (8.97)
	EMDR + TAU	66.71 (6.01)	58.79 (12.91)


*Standard deviations are given in parentheses. N_*TA*U_ = 16, N_*EMDR*__+__*TAU*_ = 14.*

**FIGURE 1:**
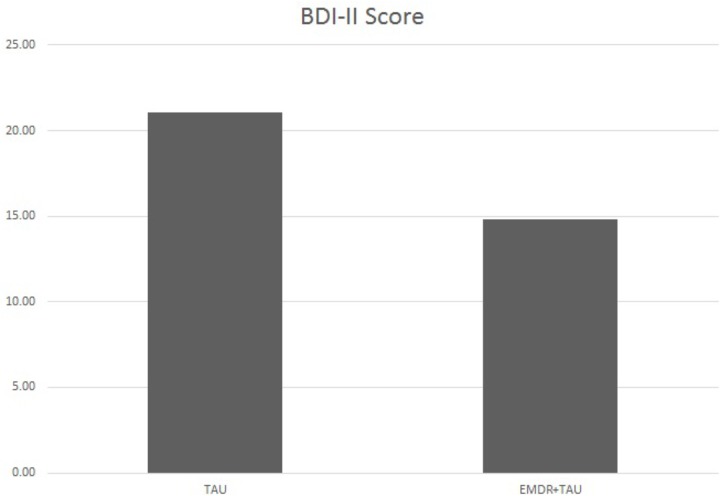
Adjusted mean BDI-II scores for TAU and EMDR + TAU at end of treatment.

**FIGURE 2:**
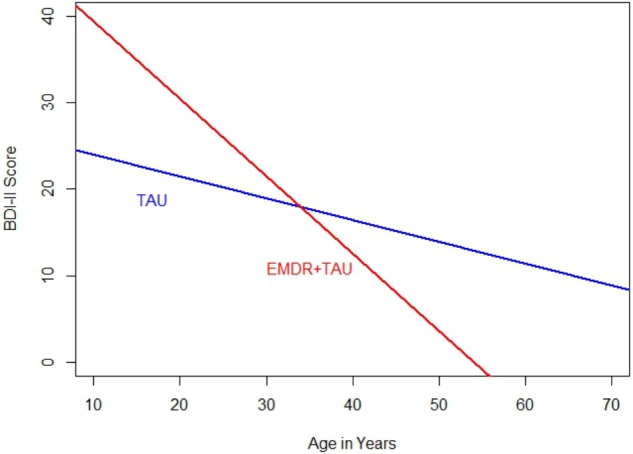
Comparison of regression slopes for age and BDI-II (end of treatment) between the two treatment groups.

**Table 3** presents descriptive statistics and correlations between variables of interest. The distribution of single and recurrent depressive episodes was not significantly different between TAU and EMDR + TAU (Fisher’s exact test: *p* = 0.68). **Table 4** presents the results of the ANCOVA of BDI-II scores at the end of treatment. The analysis controlled for the type of depression (single versus recurrent episode), patient age, total number of days in treatment, and BDI-II scores at the beginning of treatment. A significant effect of treatment group [*F*(1,21) = 6.30, *p* < 0.05, $\eta_{p}^{2}$ = 0.23] was examined by a simple contrast, which showed that the EMDR + TAU group scored significantly lower than the TAU group on adjusted end of treatment BDI-II scores (contrast value = 74.97, *p* = 0.02, $\eta_{p}^{2}$ = 0.23). **Figure 1** illustrates this contrast. Furthermore, a significant covariate effect was found for BDI-II scores at the beginning of treatment [*F*(1,21) = 8.85, *p* < 0.05, $\eta_{p}^{2}$ = 0.30]. Additionally, a significant interaction between treatment group and patient age was found [*F*(1,21) = 6.40, *p* < 0.05, $\eta_{p}^{2}$ = 0.23]. This interaction can be interpreted as the difference between EMDR + TAU and TAU concerning the magnitude of the association between age and end-of-treatment BDI-II scores. Precisely speaking, the association between patient age and end-of-treatment BDI-II scores is more positive in the TAU group than in the EMDR + TAU group. It is presented in **Figure 2**.

**Table 3:** Descriptive statistics and correlation matrix.

	*M*	*SD*	1.	2.	3.	4.	5.	6.	7.	8.
1. BDI-II (Beginning)	22.74	7.22								
2. BDI-II (End)	14.55	11.32	0.58^∗∗^							
3. SCL-90-R depression subscale (Beginning)	71.00	7.32	0.62^∗∗∗^	0.68^∗∗∗^						
4. SCL-90-R depression subscale (End)	62.48	11.72	0.55^∗∗^	0.88^∗∗∗^	0.73^∗∗∗^					
5. SCL-90-R GSI (Beginning)	68.80	6.22	0.59^∗∗^	0.64^∗∗∗^	0.85^∗∗∗^	0.62^∗∗∗^				
6. SCL-90-R GSI (End)	60.66	11.00	0.59^∗∗^	0.86^∗∗∗^	0.74^∗∗∗^	0.95^∗∗∗^	0.71^∗∗∗^			
7. Treatment group			-0.04	-0.20	-0.16	-0.23	-0.32	-0.17		
8. Age	39.74	9.52	-0.49^∗∗^	-0.45^∗^	-0.36^∗^	-0.53^∗∗^	-0.20	-0.48^∗∗^	0.06	
9. Total number of days in treatment	53.97	16.34	0.52^∗∗^	0.14	0.15	0.29	0.03	0.31	0.33	-0.33

*N = 30, M = Mean, SD = Standard deviation. Significance denoted by ^∗^p < 0.05; ^∗∗^p < 0.01; ^∗∗∗^p < 0.001.*

**Table 4:** ANCOVA of BDI-II (End).

Source	Mean square	*F*	Significance	$\eta_{p}^{2}$
Treatment group	456.43	6.30	0.02	0.23
Type of diagnosis	100.91	13.44	0.24	0.95
Age	89.68	1.22	0.28	0.06
Total number of days in treatment	50.75	0.69	0.42	0.03
BDI-II (Beginning)	650.58	8.85	0.01	0.30
Treatment group^∗^age	470.47	6.40	0.02	0.23
Treatment group^∗^total number of days in treatment	291.36	3.96	0.06	0.16
Treatment group^∗^type of diagnosis	8.67	0.12	0.74	0.01

*N = 30.*

**FIGURE 3:**
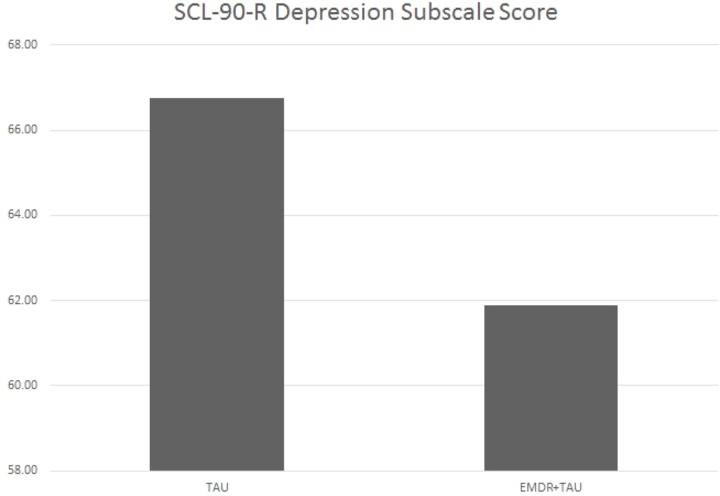
Adjusted mean SCL-90-R depression subscale scores for TAU and EMDR + TAU at end of treatment.

**Table 5** displays the results of the ANCOVA of SCL-90-R depression subscale scores at the end of treatment. The analysis controlled for the type of depression, patient age, total number of days in treatment, and SCL-90-R depression subscale scores at the beginning of treatment. A marginally significant effect for treatment group [*F*(1,20.87) = 3.44, *p* = 0.08, $\eta_{p}^{2}$ = 0.14] was examined by a simple contrast, which showed that the EMDR + TAU group had marginally significantly lower end-of-treatment SCL-90-R depression subscale scores than the TAU group (contrast value = 46.02, *p* = 0.08, $\eta_{p}^{2}$ = 0.15). **Figure 3** illustrates this contrast.

**Table 5:** ANCOVA of SCL-90-R depression subscale (End).

Source	Mean square	*F*	Significance	$\eta_{p}^{2}$
Treatment group	143.37	3.44	0.08	0.14
Type of diagnosis	112.60	1.57	0.41	0.58
Age	233.34	5.66	0.03	0.22
Total number of days in treatment	98.34	2.38	0.14	0.11
SCL-90-R depression subscale (Beginning)	553.21	13.41	<0.01	0.40
Treatment group^∗^age	279.73	6.78	0.02	0.25
Treatment group^∗^total number of days in treatment	41.62	1.01	0.33	0.05
Treatment group^∗^Type of diagnosis	75.31	1.83	0.19	0.08

*N = 29.*

**FIGURE 4:**
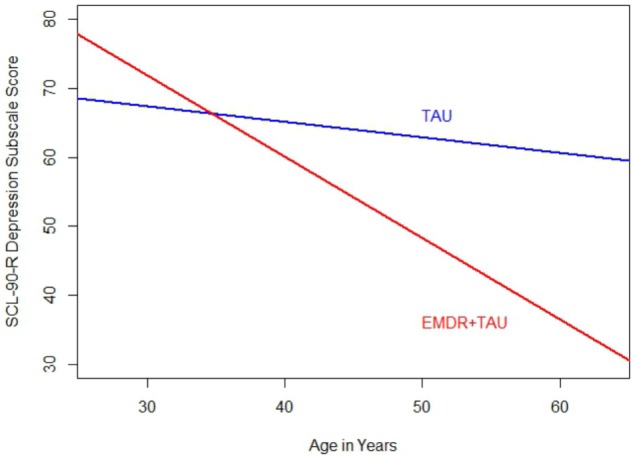
Comparison of regression slopes for age and SCL-90-R depression subscale (end of treatment) between the two treatment groups.

Moreover, significant covariate effects for patient age [*F*(1,20) = 5.66, *p* < 0.05, $\eta_{p}^{2}$ = 0.22] and beginning-of-treatment SCL-90-R depression subscale scores were found [*F*(1,20) = 13.41, *p* < 0.01, $\eta_{p}^{2}$ = 0.40]. Apart from that, a significant interaction between treatment group and patient age was found [*F*(1,20) = 6.78, *p* < 0.05, $\eta_{p}^{2}$ = 0.25]. This interaction can be interpreted as the difference in magnitude of the association between patient age and beginning-of-treatment SCL-90-R depression subscale scores. After examining the respective coefficient, it emerged that the association between patient age and SCL-90-R depression subscale scores was more positive in the TAU group than in the EMDR + TAU group. **Figure 4** illustrates this.

**FIGURE 5:**
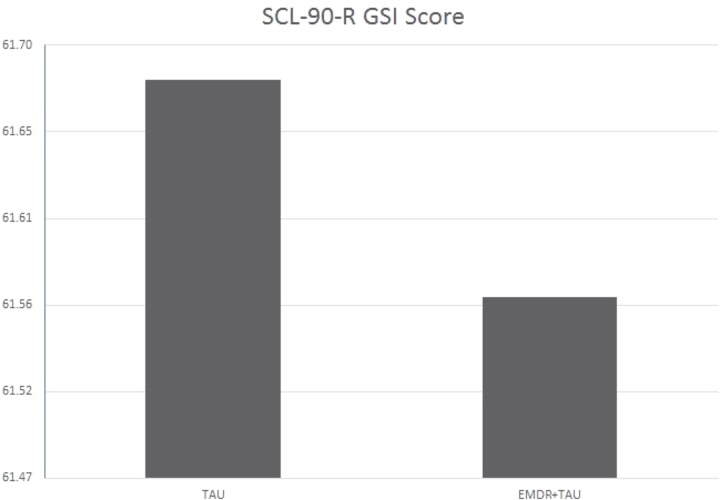
Adjusted mean SCL-90-R GSI scores for TAU and EMDR + TAU at end of treatment.

**FIGURE 6:**
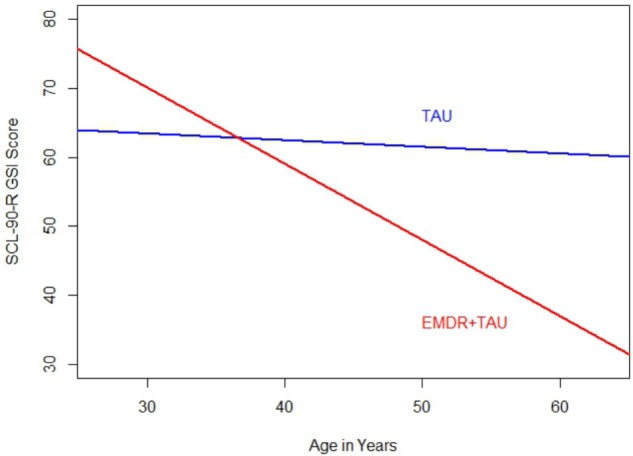
Comparison of regression slopes for age and SCL-90-R GSI scores (end of treatment) between the two treatment groups.

**Table 6** displays the results of the ANCOVA of SCL-90-R GSI scores at the end of treatment. The analysis controlled for the type of depression, patient age, total number of days in treatment, and SCL-90-R GSI scores at the beginning of treatment. A significant effect for treatment group [*F*(1,20.95) = 4.37, *p* < 0.05, $\eta_{p}^{2}$ = 0.17] was examined by a simple contrast, which showed that the EMDR + TAU group scored significantly lower on the SCL-90-R GSI than the TAU group (contrast value = 47.47, *p* < 0.05, $\eta_{p}^{2}$ = 0.18). **Figure 5** displays this graphically. Moreover, significant covariate effects for patient age [*F*(1,20) = 6.27, *p* < 0.05, $\eta_{p}^{2}$ = 0.24] and beginning-of-treatment GSI scores were found [*F*(1,20) = 21.04, *p* < 0.001, $\eta_{p}^{2}$ = 0.51]. Apart from that, a significant interaction effect between treatment group and patient age was found [*F*(1,20) = 8.00, *p* < 0.05, $\eta_{p}^{2}$ = 0.29]. This interaction can be interpreted as the difference in magnitude of the association between patient age and beginning-of-treatment SCL-90-R GSI scores. After examining its coefficient, it turned out that the association between patient age and SCL-90-R GSI scores was more positive in the TAU group than in the EMDR + TAU group. **Figure 6** illustrates this.

**Table 6:** ANCOVA of SCL-90-R GSI scores (End).

Source	Mean square	*F*	Significance	$\eta_{p}^{2}$
Treatment group	161.93	4.37	0.05	0.17
Type of diagnosis	17.63	0.21	0.72	0.16
Age	227.62	6.27	0.02	0.24
Total number of days in treatment	80.21	2.21	0.15	0.10
GSI (Beginning)	764.16	21.04	<0.001	0.51
Treatment group^∗^Age	290.46	8.00	0.01	0.29
Treatment group^∗^total number of days in treatment	28.58	0.79	0.39	0.04
Treatment group^∗^type of diagnosis	89.62	2.47	0.13	0.11

*N = 29. Scores were rounded to two decimals. The exact significance value for treatment group was below 0.05 (p = 0.048).*

The results consisted of the changes between the beginning and the end of the treatment regarding psychological tests (BDI-II, SCL-90-R depression subscale, and SCL-90-R GSI). In the EMDR + TAU group, a relatively more clear improvement compared to the TAU group showed. In seven of the 14 patients in the EMDR + TAU group, the BDI-II score dropped below nine points, falling within the normal range and being considered a full remission. In four patients, a clear improvement showed with scores dropping below 20, which is considered a slight depressive symptomatology. One patient showed mild improvement, remaining in the range of moderate depressive symptoms. In two patients, no improvements showed according to the BDI-II.

Of the 16 TAU group patients, four patients improved below nine points on the BDI-II, which can be considered a full remission. In five patients, an improvement showed, letting their scores drop below 14 into in the range of a minimally depressive symptomatology. In two patients, an improvement showed that put them in the mildly depressive symptom range (below 20 points). One patient improved to fall within 20 to 28 points, which classifies as moderately severe depressive symptomatology. Two patients remained without improvement in the severely depressed range with scores higher than 29. In two patients, the BDI-II worsened from the moderately severe to the severe range (over 29 points).

This means that 50% of the 14 patients who received EMDR + TAU showed a complete remission at the end of treatment. In the TAU group with 16 patients, only 25% of scores indicated a complete remission. The EMDR + TAU group thus showed a greater reduction of depressive symptoms than the TAU group, exhibiting significantly lower BDI-II scores at the end of treatment (see **Figure 1**). Furthermore, a significant interaction between treatment group and patient age was found. The effect indicated age was more strongly negatively related with end-of-treatment BDI-II scores in the EMDR + TAU group than in the TAU group. This suggests that older people may have benefited more from EMDR treatment than younger people.

On the SCL-90-R depression subscale, the EMDR + TAU group also showed lower end-of-treatment scores than the TAU group. In the EMDR + TAU group, 12 out of 14 patients showed a mild to marked improvement of those scores. One patient showed a mild worsening, and one patient scored the same as at the beginning of treatment. In the TAU group, 11 patients showed a mild to marked improvement. In three patients, no improvement showed relative to their scores at the beginning of treatment. One patient in this group missed this testing session. Hence, the EMDR + TAU group showed a somewhat greater reduction of depressive symptoms on the SCL-90-R depression subscale than the TAU group, albeit only marginally statistically significant in the ANCOVA analysis. This difference is shown in **Figure 3**.

An interaction effect similar to the one found in the BDI-II score analysis showed on the SCL-90-R depression subscale. It involved patient age and treatment group, indicating that higher age was more strongly associated with lower SCL-90-R depression subscale scores in the EMDR + TAU group than in the TAU group.

In the SCL-90-R GSI score analysis, the EMDR + TAU group (14 patients) showed mild to marked improvements at the end of treatment in 13 patients and a worsening of global symptom severity in one patient. In the TAU group with 16 patients, 13 patients showed mild to marked improvements, as measured by lower SCL-90-R GSI scores at the end of treatment. In two patients, stagnation showed with scores remaining unchanged from beginning to end of treatment. One patient missed the final testing, leading to missing data on this outcome.

Both groups thus showed improvements in SCL-90-R GSI scores from the beginning to the end of treatment. Like on the BDI-II, the SCL-90-R GSI scores in the EMDR + TAU group were lower than in the TAU group. Similar to the BDI-II and SCL-90-R depression subscale analyses, an interaction emerged in the analysis of SCL-90-R GSI scores between age and treatment group. This effect indicated that the negative relationship between age and end-of-treatment SCL-90-R GSI scores was stronger in the EMDR + TAU group than in the TAU group.

No side effects were reported during the treatment in the context of the study. This indicates that the EMDR treatment was well tolerated by the patients. Hyperarousal was hardly observed in the sessions. Intense affect was experienced in some sessions, but could be stabilized and reprocessed. The time frame of a maximum of 60 min per session was sufficient to process most of the treated stressful memories.

## Discussion

The present study is embedded in the larger context of the EDEN multicenter study, which investigates whether EMDR treatment has a beneficial effect in the treatment of depression. Moreover, the collection of catamnesis data helps to examine whether EMDR may reduce the number of relapses. This research is necessary due to the high worldwide prevalence of depressive disorders and the not yet satisfactory outcomes in the treatment of depression that are characterized by high relapse rates. Furthermore, the present study is relevant because it represents the first study of higher methodological quality regarding this topic, reporting on a randomized-controlled clinical trial. In order to provide more homogenous treatment conditions, the use of antidepressant medication was an inclusion criterion in the study, leading to a more naturalistic sample.

The results of this study show that patients suffering from depression benefit from adjunctive EMDR in the acute depression treatment. In the experimental group (EMDR + TAU), there was a significantly better improvement of BDI-II scores than in the control group (TAU only). Of 14 patients in the experimental group, the BDI-II score of seven patients improved below nine points, which equals a complete remission. This compares to four patients improving below nine points in the control group with 16 patients. The experimental group also showed better results on the SCL-90-R depression subscale. A mild to clear improvement was shown in 12 out of 14 patients. In the slightly larger control group, mild to clear improvements showed in 11 patients. Finally, the SCL-90-R GSI scores also showed a clearly more positive result in the experimental group than in the control group. In the experimental group, 13 patients showed mild to clear improvements. In the control group, mild to clear improvements showed for 12 patients.

The interaction between treatment group and patient age that was observed on all outcome variables (BDI-II, SCL-90-R depression subscale, and SCL-90-R GSI) showed that there was a greater age effect in the experimental group than in the control group, meaning that older patients tended to have relatively lower symptom scores than younger patients in the experimental group than in the control group. Possible explanations for this could be the greater life experience of older patients, the decreasing number of foreign missions for soldiers as they get older, the often higher rank of older soldiers within the armed forces, or the proximity to retirement. Regarding this, it would be interesting to compare this sample with patients from a different health care provider (e.g., a public health insurance provider).

The model of AIP (Shapiro, 2001) offers a potential explanation for the beneficial effects of EMDR therapy observed in the present study. The AIP model postulates that stressful events may be dysfunctionally stored and that these stressful memories may consequently form the basis of mental disorders such as depression. This means that even non-A criterion types of stressful memories can be dysfunctionally stored in memory networks. It also postulates that in reprocessing patients’ dysfunctionally stored stressful memories, they ultimately get adaptively integrated into memory networks. Many of these stressful memories in depressive disorders were memories of losses, separations, or humiliations, but also experiences of childhood emotional abuse and neglect, which are typical forms of stressful memories that appear to be related with the occurrence of depressive disorders (Mandelli et al., 2015). This fits well with studies that showed that victims of adverse life events do not remember A criterion events as more traumatic than other adverse life events (Gold et al., 2005) or in other terms, that the so-called type A criterion events were not perceived as more stressful than the so-called non-type A criterion events. In summary, the AIP model suggests a profound effect of EMDR therapy due to the processing of pathogenic memories, as described by Centonze et al. (2005).

The present study contributes to the literature not only by showing the beneficial effect of EMDR in the treatment of depression, but also by corroborating previous findings with a stronger research design. For example, compared with a previous matched-pair study (Hase et al., 2015), the present study was advantageous with regard to the randomized-controlled design. Furthermore, a disadvantage of the matched-pair study was that the BDI-II tests were only given to the 11 patients of the experimental group. This precluded the comparison of both groups regarding the rate of complete remissions, as the BDI-II tests were not given to the control group due to limited resources. Thus, the study was unable to make a scientific comparison and could only hint at the effectiveness of adjunctive EMDR treatment of depressive patients. The somewhat older, similar study of Hofmann et al. (2014) did not randomize the sample, either. It may also have been limited by the limited clinical experience of the psychotherapists in both groups and the fact that the control group consisted of patients who received CBT in the same clinic at the same time, but did not constitute a randomized treatment group. A further possible disadvantage was the unequally distributed use of antidepressants in the patients of the study. In the control group, 6 of 21 patients received antidepressants, while nine of 21 in the experimental group received antidepressant medication. The literature around EMDR therapy in the treatment of depression is likely to be strengthened by further studies using strong methodologies to examine the effect of EMDR in the treatment of depression in the context of the EDEN multicenter study.

There are several limitations to the present study. First, its low sample size limits the generalizability of the results and requires replication in order to see whether the present findings would show again in a larger sample, for example, in a multicenter study comparison, which is also planned for the EDEN study. Second, this study sampled a population of patients that were insured by the armed forces, leading to an over-representation of men. In order to account for this limitation and include more female participants, future research could sample patients insured by health care companies other than those exclusively working with military personnel. A third limitation concerns the fact that patients self-reported the severity of their depressive symptoms. More objective measures, or independent observer ratings of depressive symptoms could have strengthened the findings of this study.

In future research, one could study the efficacy of EMDR in the treatment of depression without concomitant antidepressant medication in an outpatient sample. This would be possible in mildly to moderately depressed patients. Furthermore, the expected meta-analysis of the EDEN multicenter study remains a prospect for the further scientific investigation of EMDR therapy in the treatment of depressive disorders. This will also show whether the positive effects of EMDR found in the present study can be supported in a greater population. Lastly, one could still examine the hypothesized beneficial effect of EMDR therapy in reducing the depressive relapse rate at follow-up.

## Conclusion

Given the predicted worldwide increase of depression and the limited success of TAU, it is important to develop adjunctive therapy strategies. The present randomized study examined whether EMDR therapy produces a positive effect in the treatment of depression beyond TAU. On the BDI-II and the GSI score of the SCL-90-R, additional EMDR treatment produced significant improvements over the effects of TAU, while it produced marginally significant improvements over TAU on the depression subscale of the SCL-90-R. Given the previously high rate of non-responders to TAU, the present study thus suggests that EMDR may improve treatment outcomes when added to TAU. The present study significantly contributes to the knowledge base in the field as it is the first to have used a randomized-controlled study design to examine the efficacy of EMDR in the treatment of depression. However, its low sample size reduces the generalizability of the results and calls for larger future studies to replicate the effects found in this study. Follow-up comparisons to the present study will reveal whether adjunctive EMDR therapy also produced more sustainable treatment effects as manifested by fewer depressive relapses at follow-up.

## Author Contributions

MH and JP acted as center and data manager in the study and share first authorship. AdH contributed the statistical analysis. LO and ArH acted as study manager and internal reviewers. RB assisted in participant recruitment and further contributed by reviewing the literature. CH took responsibility as senior author.

## Conflict of Interest Statement

MH and ArH offer education in EMDR therapy for licensed psychotherapists. The remaining authors declare that the research was conducted in the absence of any commercial or financial relationships that could be construed as a potential conflict of interest.
